# Narrowing the Phase Distribution of Quasi‐2D Perovskites for Stable Deep‐Blue Electroluminescence

**DOI:** 10.1002/advs.202201807

**Published:** 2022-07-06

**Authors:** Yoonseo Nah, Devan Solanki, Yitong Dong, Jason A. Röhr, André D. Taylor, Shu Hu, Edward H. Sargent, Dong Ha Kim

**Affiliations:** ^1^ Division of Chemical Engineering and Materials Science College of Engineering Ewha Womans University 52 Ewhayeodae‐gil, Seodaemun‐gu Seoul 03760 Republic of Korea; ^2^ Department of Chemical and Environmental Engineering Yale University New Haven CT 06511 USA; ^3^ Energy Sciences Institute Yale University West Haven CT 06516 USA; ^4^ Department of Electrical and Computer Engineering University of Toronto 10 King's College Road Toronto Ontario M5S 3G4 Canada; ^5^ Department of Chemistry and Biochemistry The University of Oklahoma Norman OK 73019 USA; ^6^ Department of Chemical and Biomolecular Engineering Tandon School of Engineering New York University Brooklyn NY 11201 USA; ^7^ Department of Chemistry and Nano Science Division of Molecular and Life Sciences College of Natural Sciences Ewha Womans University 52, Ewhayeodae‐gil, Seodaemun‐gu Seoul 03760 Republic of Korea; ^8^ Basic Sciences Research Institute (Priority Research Institute) Ewha Womans University 52, Ewhayeodae‐gil, Seodaemun‐gu Seoul 03760 Republic of Korea; ^9^ Nanobio∙Energy Materials Center (National Research Facilities and Equipment Center) Ewha Womans University 52, Ewhayeodae‐gil, Seodaemun‐gu Seoul 03760 Republic of Korea

**Keywords:** antisolvent engineering, blue light‐emitting diodes, deep‐blue electroluminescence, energy landscape, evaporation kinetics, quantum well dispersity, quasi‐2D perovskites

## Abstract

Solution‐processed quasi‐2D perovskites contain multiple quantum wells with a broad width distribution. Inhomogeneity results in the charge funneling into the smallest bandgap components, which hinders deep‐blue emission and accelerates Auger recombination. Here, a synthetic strategy applied to a range of quasi‐2D perovskite systems is reported, that significantly narrows the quantum well dispersity. It is shown that the phase distribution in the perovskite film is significantly narrowed with controlled, simultaneous evaporation of solvent and antisolvent. Modulation of film formation kinetics of quasi‐2D perovskite enables stable deep‐blue electroluminescence with a peak emission wavelength of 466 nm and a narrow linewidth of 14 nm. Light emitting diodes using the perovskite film show a maximum luminance of 280 cd m^–2^ at an external quantum efficiency of 0.1%. This synthetic approach will serve in producing new materials widening the color gamut of next‐generation displays.

## Graphical abstract

Modulation of film formation kinetics has paved the way for developing deep‐blue emissive quasi‐2D perovskites. A universal synthetic strategy, potentially applicable to various material systems, is introduced to narrow the width distribution of perovskite quantum wells. The controlled and simultaneous evaporation of solvent and antisolvent is key.

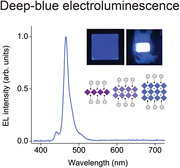

## 1 Introduction

Quasi‐2D perovskites consist of nanometer‐thick halide perovskites separated by organic spacers. They have recently been considered as candidate light‐emitters since the dielectric confinement provides a large exciton binding energy,^[^ ^1^, ^2^, ^3^ ^]^ their defect tolerance allows superior color purity,^[^ ^4^ ^]^ and they are solution‐processible at room temperature.^[^ ^5^ ^]^

Solution‐processed quasi‐2D perovskites contain multiple quantum wells and often exhibit a broad width distribution. Inhomogeneity derives from spontaneous formation of colloidal sol‐gel complexes, which offer nucleation sites for local crystallization.^[^ ^6^, ^7^ ^]^ As a result, each nanocrystalline slab with its unique bandgap energy orients randomly with respect to the substrate.

Inhomogeneous band alignment allows charge carriers to be funneled into the lowest‐energy radiative‐recombination centers and saturate shallow trap sites.^[^ ^5^, ^8^, ^9^ ^]^ This efficient energy funneling contributes to increased photoluminescence quantum yield.^[^ ^9^, ^10^, ^11^ ^]^ However, the resulting cascade energy landscape often limits device performance in two ways: (i) excitons recombine in large *n* phases or quasi‐3D phases, thus hindering the deep‐blue emission; and (ii) Auger recombination dominates at high current density due to the increased charge carriers confined in a small subpopulation of recombination centers.^[^ ^12^, ^13^ ^]^

To overcome these limitations, it is important to control quantum well dispersity. A narrow phase distribution allows deep‐blue electroluminescence from small *n* phases and also mitigates Auger recombination, since it increases the number of recombination centers. Given the difficulty in controlling the crystallization rate during the sol‐gel stage, however, only a few studies have reported narrow quantum well distributions to date.^[^ ^14^, ^15^, ^16^, ^17^, ^18^ ^]^ Additionally, the most promising strategies currently are based on modulating chemical composition; this limits material systems and often relies on a multistep synthesis.

**Figure 1:**
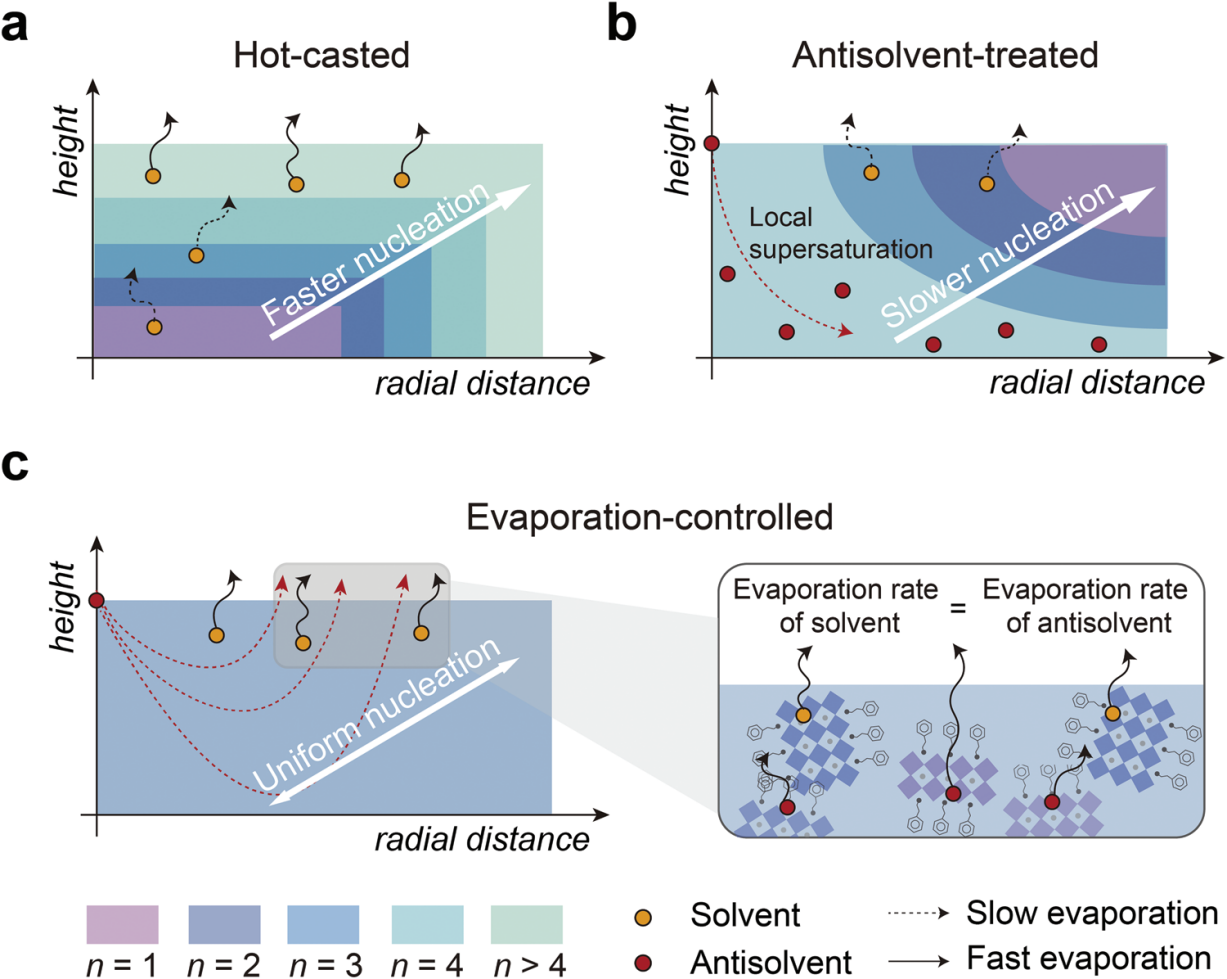
Schematic diagrams illustrating the phase distribution of <*n*> = 2 perovskites synthesized using different techniques. a) Hot‐casted perovskite showing vertical and lateral phase segregation, as the hot‐casting method accelerates the evaporation of solvent at the liquid–air interface. b) Antisolvent treatment also leads to the spatially non‐uniform nucleation, because antisolvent molecules spread above the substrate and reduce local solubility. Consequently, hot‐casted and antisolvent‐treated quasi‐2D perovskites contain multiple quantum wells with a broad width distribution. c) In contrast, when the evaporation rate of solvent at the liquid–air interface equates with the evaporation rate of antisolvent near the substrate, the supersaturated regions are minimized and perovskites crystallize at a uniform nucleation rate. Therefore, the evaporation‐controlled perovskite shows the narrowest phase distribution.

Here we use film formation kinetics to tailor the phase dispersity of various Ruddlesden–Popper phase perovskite materials. Prior reports showed that solvent‐precursor intermediates promote the growth of quantum wells, as solvent evaporation releases inorganic precursors and incorporates these into partially formed quasi‐2D slabs.^[^ ^19^, ^20^ ^]^ The thickness of quantum wells is thus determined after the formation of organic bilayers. We note that crystallization kinetics can be tailored by modulating the synthesis parameters. For example, in light of the rapid evaporation of solvent at the liquid–air interface, the local supersaturation at this regime is generally higher than that at the liquid–substrate interface. Therefore, hot‐casted films show a gradual increase in slab thickness (*n* value) along the vertical and lateral directions (**Figure** **1**a).^[^ ^21^ ^]^ In contrast, the phase distribution in antisolvent‐treated perovskite film is different due to the higher local supersaturation near the substrate, which results from the uneven distribution of antisolvent molecules as depicted in Figure 1b. Whereas thick quantum wells crystallize near the substrate due to the prompt local supersaturation, the combination of slower nucleation and an excess amount of residual organic spacers leads to the formation of thinner wells on the corner regions.

## 2 Results and Discussion

To narrow the phase distribution, we sought to prolong the process of incorporation of newly released precursors into the existing perovskite slabs. This can be achieved either by (i) accelerating the formation of organic bilayers to quench the growth of quantum wells or (ii) regulating the nucleation rates throughout the film. Assuming a uniform distribution of dissolved organic moieties in the film and their low diffusivity due to their large sizes, a viable approach to accelerate the formation of organic bilayers is to increase their concentration. However, employing more organic spacers than the stoichiometric quantity leads to more monolayered slabs, which decreases the charge transport capability.^[^ ^22^ ^]^

Alternatively, spatially uniform nucleation also restricts the growth of thick quantum wells as it implies an increased number of nucleation sites. Considering that the evaporation of solvent starts from the liquid–air interface and that the antisolvent lingers near the substrate, uniform nucleation can be achieved by matching the temperature of the system (i.e., precursor solution and substrate) with the boiling point of antisolvent (Figure 1c). This will induce simultaneous evaporation of solvent and antisolvent, which accelerates nucleation throughout the film and enables even distribution of organic barriers. The width distribution of perovskite quantum wells can thus be regulated.

To verify our proposed approach, we synthesized quasi‐2D perovskites (PEA_2_CsPb_2_Br_7_, *n* = 2) on glass substrates by using three different techniques: (i) hot‐casting, (ii) antisolvent‐treatment, and (iii) evaporation‐control methods. First, precursor solutions were prepared by dissolving stoichiometric amounts of 2‐phenylethylammonium bromide (PEABr), cesium bromide (CsBr), and lead bromide (PbBr_2_) into dimethylsulfoxide (DMSO). Small amounts of *n*‐propylammonium bromide (nPABr) were added to improve crystallinity and to suppress the formation of the *n* = 1 phase.^[^ ^23^, ^24^, ^25^ ^]^ This nPABr has negligible impact on the formation of larger *n* phases (see Figure S3, Supporting Information). For the syntheses of hot‐casted and evaporation‐controlled perovskites, the precursor solutions and the glass substrates were pre‐heated at 110 ℃ for 20 min and then transferred to a spin‐coater. Meanwhile, during the spin‐coating process of antisolvent‐treated and evaporation‐controlled perovskites, 150 µL of toluene was dropped onto the substrate (see Experimental Section).

**Figure 2:**
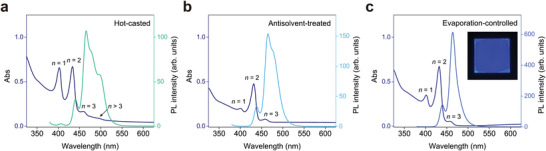
Photophysical characterizations. a–c) UV–vis absorption and photoluminescence spectra of <*n*> = 2 perovskites (PEA_2_CsPb_2_Br_7_) synthesized using different techniques. The excitation wavelength was 365 nm. The inset photograph was taken under a UV lamp. Film size: 25 × 25 mm.

We first compared the photophysical properties of <*n*> = 2 perovskites synthesized using different techniques. As shown in **Figure** **2**a, the hot‐casted perovskite exhibits sharp excitonic absorption peaks which correspond to the absorption from *n* = 1, 2, 3 and quasi‐3D phases. Multiple emission peaks indicate inefficient energy transfer from larger to smaller bandgap components. On the contrary, the antisolvent‐treated perovskite exhibits reduced absorption from *n* > 3 phases, suggesting a reduction of the thicker *n* > 3 slabs in the film (Figure 2b). It should be noted, however, that this does not preclude the existence of large *n* phases. The photoluminescence spectrum shows a distinct shoulder at 477 nm, which implies that a considerable amount of photon energy is delivered to *n* > 3 phases despite their small concentration. In stark contrast, the quasi‐2D perovskite synthesized under uniform evaporation does not exhibit any absorption nor emission peaks that correspond to *n* > 3 phases (Figure 2c). Although emission from the *n* = 2 phase is still observable, the sharp fluorescence peak at 464 nm with full‐width at half‐maximum (FWHM) of 19 nm confirms that the maximum *n* value of existing inorganic frameworks is 3.

We then sought to investigate the spatial distributions of quantum wells by measuring the photoluminescence spectra at the center and corner parts of each film (see Figure S1, Supporting Information). Unlike previous measurements in which excitation laser beams were sent onto the front side of perovskite layers, films were illuminated from the back side under various excitation wavelengths; fluorescence spectra measured with shorter excitation wavelengths, for example, would provide information about energy landscape near the substrate due to the short penetration depths.^[^ ^28^ ^]^ Yet, considering that emission peak intensities of multiple quantum well systems depend on many factors (including quantum well alignment, exciton funneling, and photon reabsorption), these spectra should be carefully examined. As expected, the hot‐casted perovskite shows a smaller degree of vertical phase segregation at the corner where surface‐area‐to‐volume ratio is larger than that of the central region (Figure S1e,h, Supporting Information). Antisolvent‐treated perovskite, however, shows the opposite trend (Figure S1f,i, Supporting Information). A notable increase in emission peak intensities of small *n* slabs at the corner under increasing excitation wavelengths indicates a higher degree of phase segregation and that thinner quantum wells dominate near the perovskite–air interface. Overall, evaporation‐controlled perovskite has the smallest degree of vertical and lateral phase segregation, which confirms that simultaneous evaporation of solvent and antisolvent allows uniform nucleation rates throughout the film.

Note that precise control of the temperature is crucial to achieve the spatially uniform nucleation. A broad absorption tail emerges when the temperature of the system is 20 ℃, which is significantly lower than the boiling point of the antisolvent toluene (110.6 ℃) (see Figure S4, Supporting Information). Interestingly, a similar trend is observed when the temperature of the system is significantly higher than that of the antisolvent (155 ℃). These results imply that toluene should evaporate rapidly to narrow the phase distribution, yet should linger in DMSO for a sufficient amount of time. This assumption was corroborated by performing control experiments using different antisolvents. As presented in Figure S5 (see Supporting Information), when the boiling point of the antisolvent is higher (chlorobenzene, 132 ℃) or lower (chloroform, 61.2 ℃) than the temperature of the system (110 ℃), the synthesized perovskites will contain large *n* phases. These results indicate that the narrowest phase distribution is achieved when the temperature of the system is comparable to the boiling point of the antisolvent.

**Figure 3:**
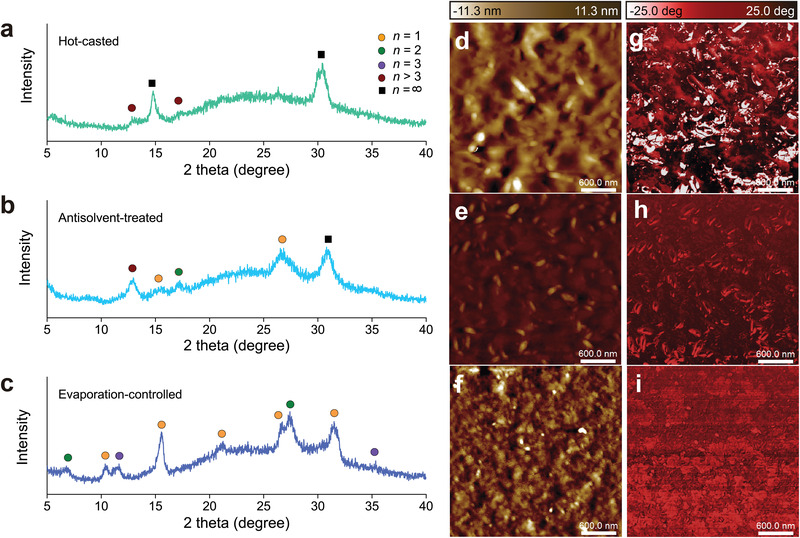
Structural characterizations. a–c) Film X‐ray diffraction patterns. Peaks were assigned by comparing with the diffraction patterns of single crystal counterparts. d–f) AFM topography. g–i) Phase contrast images of <*n*> = 2 perovskites (PEA_2_CsPb_2_Br_7_) synthesized using different techniques.

To further investigate the phase distribution of perovskite films, we performed structural analyses of <*n*> = 2 perovskites synthesized using different techniques. As shown in **Figure** **3**a, the film X‐ray diffraction (XRD) pattern of the hot‐casted perovskite shows *n* > 3 phases, which indicates that the formation of quasi‐3D slabs is dominant at the liquid–air interface. In comparison, antisolvent‐treated perovskites exhibit intense diffraction peaks that correspond to the monolayer perovskite as well as large *n* phases (Figure 3b). The lower degree of vertical phase segregation compared to the hot‐casted counterpart is attributed to the relatively slower evaporation of toluene. In contrast, the XRD pattern of the evaporation‐controlled perovskites is significantly different from that of hot‐casted and antisolvent‐treated perovskites (Figure 3c). Sharp diffraction peaks confirm enhanced crystallinity in evaporation‐controlled perovskites, implying reduced energy landscape disorder within the system. Most importantly, peaks corresponding to *n* > 3 phases are not observed, which supports the improved phase distribution using the evaporation‐controlled approach.

Atomic force microscopy (AFM) measurements corroborated the observation that the phase distribution of quasi‐2D perovskites depends on synthetic techniques. Arithmetic average values of surface roughness of the hot‐casted, antisolvent‐treated and evaporation‐controlled perovskite are 8.68, 3.08, and 1.23 nm, respectively (Figure 3d–f). We also measured phase lag between driving signal of cantilever oscillation and its output signal, which reflects the variance in mechanical properties of the surface. As presented in Figure 3g–i, hot‐casted perovskite shows the most pronounced phase contrast, whereas the evaporation‐controlled perovskite film shows smallest phase difference. Given that the elasticity of quasi‐2D perovskites is largely determined by the thickness of inorganic layers,^[^ ^29^ ^]^ larger phase contrast can be translated into a broader phase distribution. In summary, structural and photophysical characterizations indicate that the quasi‐2D perovskite synthesized using a spatially uniform evaporation approach has the narrowest width distribution of quantum wells.

**Figure 4:**
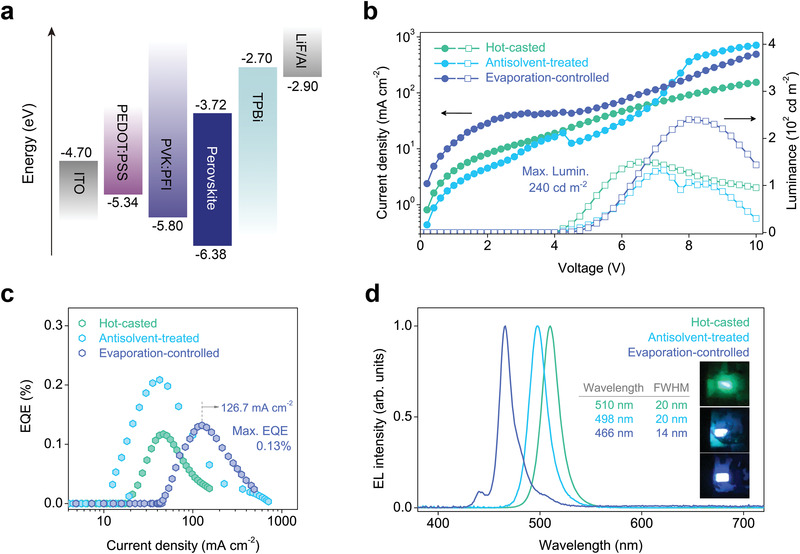
Device performance. a) Schematic diagram illustrating the configuration of tested perovskite light‐emitting diodes. b) Plots of current density and luminance as a function of driving voltage. The turn‐on voltages were determined to be 3.7 V. c) Plots of external quantum efficiency as a function of current density. d) Electroluminescence spectra of devices at a driving voltage of 7.0 V. Inset photographs were taken during the measurements (Top: hot‐casted, middle: antisolvent‐treated, bottom: evaporation‐controlled perovskite light‐emitting diodes). Device size: 3 × 2 mm.

We then fabricated multilayer electroluminescence devices and measured their performance. As illustrated in **Figure** **4**a, each device has a configuration of ITO/PEDOT:PSS (<10 nm)/PVK:PFI (≈15 nm)/perovskite/TPBi (40 nm)/LiF (2 nm)/Al (70 nm). The valence band edge (*E*_v_) of perovskite layer was determined by performing ultraviolet photoelectron spectroscopy (UPS) measurement (see Figure S6, Supporting Information), and the conduction band edge (*E*_c_) was estimated by adding the optical bandgap energy (Figure 2). Under forward bias, holes are injected from the anode to PEDOT:PSS (poly(3,4‐ethylenedioxythiophene) polystyrene sulfonate) and then transferred to PVK (poly(9‐vinylcarbazole)). At the same time, electrons are injected from the cathode to TPBi (2,2′,2′′‐(1,3,5‐benzenetriyl)tris(1‐phenyl‐1H‐benzimidazole)). Charge carriers are then transported to the perovskite layer and undergo radiative or nonradiative recombination. The electronic band structure of perovskites was determined by performing ultraviolet photoelectron spectroscopy (UPS) measurement. It should be acknowledged that the choice of hole transporting materials, i.e., the growth substrates, can affect the crystal growth dynamics of the perovskite layer.^[^ ^26^, ^27^ ^]^ While these effects are outside the scope of this study, we aim to investigate this in the future.

Performance of tested devices is summarized in **Table** **1** and Figure 4b–d. The electroluminescence peak wavelengths of the hot‐casted, antisolvent‐treated, and evaporation‐controlled perovskites locate at 510, 498, and 466 nm, respectively. None of these devices experience spectral instability, as the perovskites do not consist of mixed anions (see Figure S11 and S12, Supporting Information).^[^ ^30^ ^]^ Note that the electroluminescence spectra of hot‐casted and antisolvent‐treated perovskites are significantly different from their photoluminescence spectra, which indicates efficient charge transfer from lower *n* phases to quasi‐3D slabs (Figure 2a,b). Consequently, large *n* phases, albeit existing in a small concentration, must be eliminated to achieve deep‐blue electroluminescence. In contrast, the evaporation‐controlled perovskite shows a peak emission wavelength at 466 nm with a FWHM of 14 nm; this is the narrowest FMHM reported to date among deep‐blue quasi‐2D perovskite devices. This extremely narrow linewidth confirms suppressed energetic disorder, indicative of a homogeneous energy landscape.^[^ ^31^ ^]^

**Table 1:** Characteristics of tested devices

Synthetic technique^a)^	*λ*_EL_ [nm]^b)^	FWHM [nm]^c)^	CIE [x,y]^b)^	Voltage [V]^c)^	Luminance [cd m^–2^]^d)^	EQE [%]^e)^	Power efficiency [lm W^–1^]^f)^	Current efficiency [cd A^–1^]^g)^
Hot‐casted	510	20	(0.05, 0.63)	6.75/6.00	150.0/135.2	0.10/0.12	0.11/0.15	0.25/0.29
Antisolvent‐treated	498	20	(0.05, 0.50)	7.25/6.50	132.4/103.1	0.12/0.21	0.06/0.12	0.13/0.24
Evaporation‐controlled	466	14	(0.15, 0.10)	8.00/7.25	240.0/200.3	0.11/0.13	0.05/0.07	0.13/0.16

^a)^ Device configuration: ITO/PEDOT:PSS/PVK:PFI/PEA_2_CsPb_2_Br_7_/TPBi/LiF/Al.

^b)^ The electroluminescence peak wavelength, FWHM and CIE coordinates at the driving voltage of 7.0 V.

^c)^ The driving voltage at maximum luminance and at maximum EQE.

^d)^ Maximum luminance value and value at maximum EQE.

^e)^ External quantum efficiency value at maximum luminance and maximum EQE value.

^f)^ Power efficiency value at maximum luminance and value at maximum EQE;

^g)^ Current efficiency value at maximum luminance and value at maximum EQE.

The device based on the evaporation‐controlled perovskite shows a maximum luminance of 240 cd m^–2^, which is a record‐high value for reported quasi‐2D perovskite deep‐blue emitting LEDs emitting (see Table S1, Supporting Information). In addition, as presented in Figure 4c, hot‐casted and antisolvent‐treated perovskites suffer from severe efficiency roll‐off at low current density (<50 mA cm^–2^), which is attributed to the charge accumulation in recombination centers. In stark contrast, the efficiency of the evaporation‐controlled perovskite starts to decrease at a much higher current density (≈126.7 mA cm^–2^) despite stronger Coulomb electron–hole interactions in *n* = 3 phases. Increasing EQE values over a wider range of current densities are ascribed to an increased subpopulation of recombination centers, as a narrower phase distribution promotes a spontaneous formation of additional *n* = 3 phases. It is anticipated that device performance can be improved further by employing passivation agents and optimizing the device architecture.^[^ ^32^ ^]^

**Figure 5:**
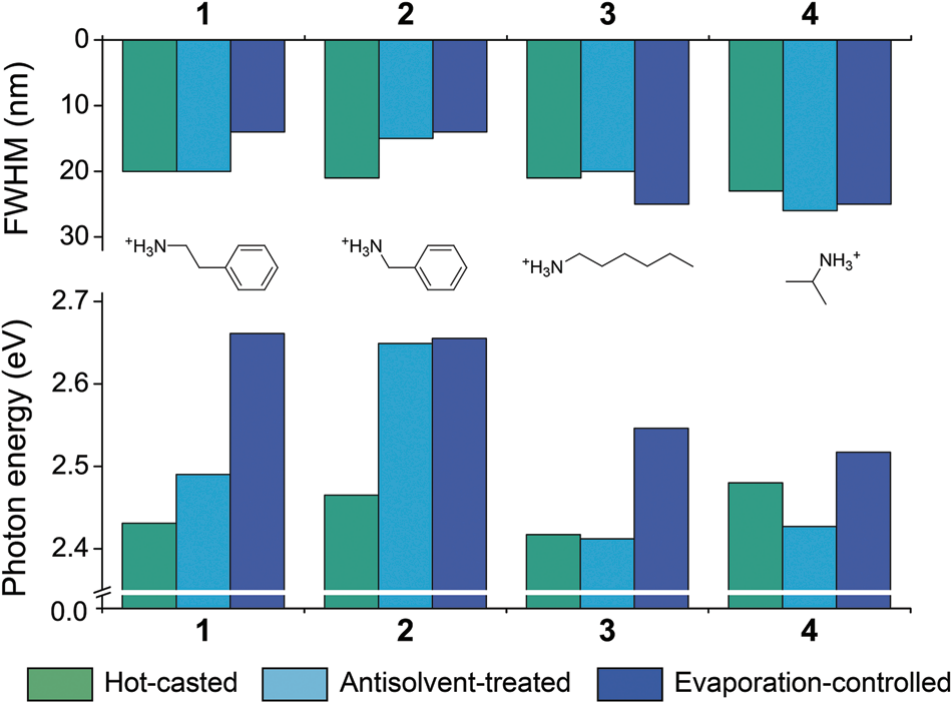
Versatility of our strategy. Full‐width at half maximum (FWHM) and photon energy values of electroluminescence peaks of <*n*> = 2 perovskites based on (1) 2‐phenylethylammonium, (2) benzylammonium, (3) *n*‐hexylammonium, and (4) isopropyl ammonium organic spacers.

We emphasize that our strategy is independent of material compositions and hence can be extended to various types of quasi‐2D perovskite systems (see Figures S7–S9, Supporting Information). As presented in **Figure** **5**, a similar trend is found for <*n*> = 2 perovskites consisting of different aromatic (benzylammonium, **2**) or aliphatic (*n*‐hexylammonium, **3**, and isopropyl ammonium, **4**) organic spacers. In all systems, evaporation‐controlled perovskites exhibit the narrowest phase distribution and emit blue electroluminescence. Our method can also be applied to iodide‐based perovskites, can therefore be used as a new strategy for achieving stable yellow emission, a long‐time challenge (see Figure S10, Supporting Information). It should be noted, however, that the formation kinetics of organic bilayers should also affect the resulting energy landscape. For example, an <*n*> = 2 perovskite based on a benzylammonium cation records a maximum luminance of 277.3 cd m^–2^ with emission peak wavelength at 467 nm (see Figure S13, Supporting Information). Interestingly, the emission peak wavelength of the corresponding perovskite that was synthesized using the antisolvent technique was similar, yet its device performance was inferior compared to its evaporation‐controlled counterpart. These results indicate rapid formation of benzylammonium bilayers, presumably due to strong *π*–*π* interactions. In contrast, recombination centers in perovskites based on aliphatic organic spacers are located at the *n* = 4 slabs. This is because the formation of organic barriers relies on weak van der Waals interactions (see Figures S14 and 15, Supporting Information). As a result, perovskites emit sky‐blue electroluminescence and show poor device performance due to the increased energetic disorder.

## 3 Conclusion

To summarize, the width distribution of perovskite quantum wells can be tailored by controlling the evaporation kinetics. Simultaneous evaporation of solvent and antisolvent enables spatially uniform nucleation of perovskite crystallites, which entails narrow phase distribution. To do so, the temperature of the system should be matched with the boiling point of the antisolvent. By performing structural and photophysical analyses, we proved that the phase dispersity of well‐studied <*n*> = 2 perovskites were significantly decreased. The resulting perovskites emitted deep‐blue electroluminescence with maximum luminance of up to 277.3 cd m^–2^, with an extremely narrow linewidth of 14 nm. Moreover, evaporation‐controlled perovskites showed maximum EQE at higher current density compared to their hot‐casted and antisolvent‐treated counterparts, which is attributed to an increased subpopulation of recombination centers. We envision our strategy could be extended to other material systems and will forge a new path to designing deep‐blue emissive quasi‐2D perovskite materials.

## 4 Experimental Section

### Materials

Lead(II) bromide (PbBr_2_), cesium bromide (CsBr), cesium iodide (CsI), isopropyl ammonium bromide (iPABr), 2‐phenylethylammonium bromide (PEABr), 2‐phenylethylammonium iodide (PEAI), dimethyl sulfoxide (DMSO), toluene, chloroform, chlorobenzene, poly(9‐vinylcarbazole) (PVK), Nafion perfluorinated resin solution (PFI), and lithium fluoride (LiF) were purchased from Sigma‐Aldrich. *n*‐propylammonium bromide (nPABr) and benzylammonium bromide (BABr) were purchased from GreatCell Solar. PEDOT:PSS Al4083 was purchased from Heraeus. 2,2′′,2′′‐(1,3,5‐benzenetriyl)tris(1‐phenyl‐1H‐benzimidazole) (TPBi) was purchased from Tokyo Chemical Industry. Al was purchased from Itasco. All commercially available chemicals were used as received without further purification.

### Fabrication of Perovskite Thin Films

Perovskite precursor solution was prepared by dissolving stoichiometric quantities of PbBr_2_, CsBr, and PEABr in DMSO solvent. The molar concentration of PbBr_2_ was fixed at 0.30 m. 10 mg mL^−1^ of nPABr was added to the precursor solution to improve the crystallinity. The resulting solution was stirred vigorously at 110 ℃ for 20 min and filtered using a polytetrafluoroethylene syringe filter (0.2 µm). For the fabrication of hot‐casted perovskite, hot precursor solution was immediately spin‐coated onto the pre‐heated substrate (110 ℃) at 4000 rpm for 20 s. For the fabrication of antisolvent‐treated perovskite, precursor solution was cooled down to the room temperature and spin‐coated at 4000 rpm for 60 s. During this process, 150 µL of toluene was dropped onto the substrate as an antisolvent. For the fabrication of evaporation‐controlled perovskite, hot precursor solution was immediately spin‐coated onto the pre‐heated substrate (110 ℃) at 4000 rpm for 20 s. During this process, 150 µL of antisolvent was dropped onto the substrate. All resulting films were then annealed at 100 ℃ for 4 min.

### Fabrication of Perovskite Light‐Emitting Diodes

The PEDOT:PSS was spin‐coated on pre‐cleaned ITO substrates at 5000 rpm for 40 s and annealed at 150 ℃ for 20 min. PVK dissolved in chlorobenzene (3 mg mL^−1^) was spin‐coated on top of the PEDOT:PSS layer at 4000 rpm for 35 s and annealed at 100 ℃ for 20 min. 0.1 wt% of PFI dissolved in isopropyl alcohol was then spin‐coated at 4000 rpm for 30 s to reduce the hole injection barrier. On top of the perovskite layer, a 40‐nm TPBi layer, a 2‐nm LiF layer, and a 70‐nm thick Al layer were deposited consecutively using a thermal evaporation system at a pressure <1.0 × 10^–6^ torr. The deposition rates of the organic and metal layers were 0.1 and 0.3 nm s^–1^, respectively. Devices were encapsulated using UV‐curable resin and transferred to a dry room for measurements.

### Steady‐State UV–Vis Absorption Measurements

UV–vis absorption spectra were collected using a Varian, Cary 5000 Spectrometer at 298 K.

### Steady‐State Photoluminescence Measurements

Steady‐state photoluminescence spectra were measured by using a JASCO, FP‐8500 spectrofluorometer at 298 K. The excitation wavelength was 365 nm.

### UV Photoelectron Spectroscopy Measurements

Photoelectron spectroscopy was performed using a ULVAC‐PHI, Veresprobe II spectroscope.

### Atomic Force Microscopy Measurements

Morphologies and phase contrast images of perovskite thin films were characterized by employing a Bruker, Dimension Edge atomic force microscope.

### Device Characterizations

Electroluminescence performance of devices were obtained by using a KEITHLEY, Keithley 2400 sourcemeter and YPCMC, CS‐2000 spectroradiometer.

## Conflict of Interest

The authors declare no conflict of interest.

## Supporting information

Supporting Information

## Data Availability

The data that support the findings of this study are available from the corresponding author upon reasonable request.
