# Supplementary material for: Narrowing the Phase Distribution of Quasi‐2D Perovskites for Stable Deep‐Blue Electroluminescence
Source: Adv Sci (Weinh). 2022 Jul 6;9(24):2201807. doi: 10.1002/advs.202201807 (PMC9404385; doi:10.1002/advs.202201807)
Supplement: Supplementary file 1 — Supporting Information [file ADVS-9-2201807-s001.pdf]

ADVANCED SCIENCE
Open Access

# Supporting Information

for *Adv. Sci.*, DOI 10.1002/advs.202201807

## Narrowing the Phase Distribution of Quasi-2D Perovskites for Stable Deep-Blue Electroluminescence

*Yoonseo Nah, Devan Solanki, Yitong Dong, Jason A. Röhr, André D. Taylor, Shu Hu, Edward H. Sargent* and Dong Ha Kim**

# Supporting Information

**Narrowing the Phase Distribution of Quasi-2D Perovskites for Stable Deep-Blue Electroluminescence**

*Yoonseo Nah, Devan Solanki, Yitong Dong, Jason A. Röhr, André D. Taylor, Shu Hu, Edward H. Sargent,* Dong Ha Kim**

Yoonseo Nah, Prof. Dong Ha Kim
Division of Chemical Engineering and Materials Science, College of Engineering, Ewha Womans University, 52 Ewhayeodae-gil, Seodaemun-gu, Seoul 03760, Republic of Korea
E-mail: dhkim@ewha.ac.kr

Yoonseo Nah, Devan Solanki, Prof. Shu Hu
Department of Chemical and Environmental Engineering, Yale University, New Haven, Connecticut 06511, United States
Energy Sciences Institute, Yale University, West Haven, Connecticut 06516, United States

Dr. Yitong Dong, Prof. Edward H. Sargent
Department of Electrical and Computer Engineering, University of Toronto, 10 King's College Road, Toronto, Ontario M5S 3G4, Canada

Dr. Yitong Dong
Department of Chemistry and Biochemistry, The University of Oklahoma, Norman, OK 73019, United States

Dr. Jason A. Röhr, Prof. André D. Taylor
Department of Chemical and Biomolecular Engineering, Tandon School of Engineering, New York University, Brooklyn, NY 11201, USA

Prof. Dong Ha Kim
Department of Chemistry and Nano Science, Division of Molecular and Life Sciences, College of Natural Sciences, Ewha Womans University, 52, Ewhayeodae-gil, Seodaemun-gu, Seoul 03760, Republic of Korea

Prof. Dong Ha Kim
Basic Sciences Research Institute (Priority Research Institute), Ewha Womans University, 52, Ewhayeodae-gil, Seodaemun-gu, Seoul 03760, Republic of Korea

Prof. Dong Ha Kim
Nanobio·Energy Materials Center (National Research Facilities and Equipment Center), Ewha Womans University, 52, Ewhayeodae-gil, Seodaemun-gu, Seoul 03760, Republic of Korea

Keywords: blue light-emitting diodes, deep-blue electroluminescence, quasi-2D perovskites, quantum well dispersity, energy landscape, antisolvent engineering, evaporation kinetics

## Supplementary Figures

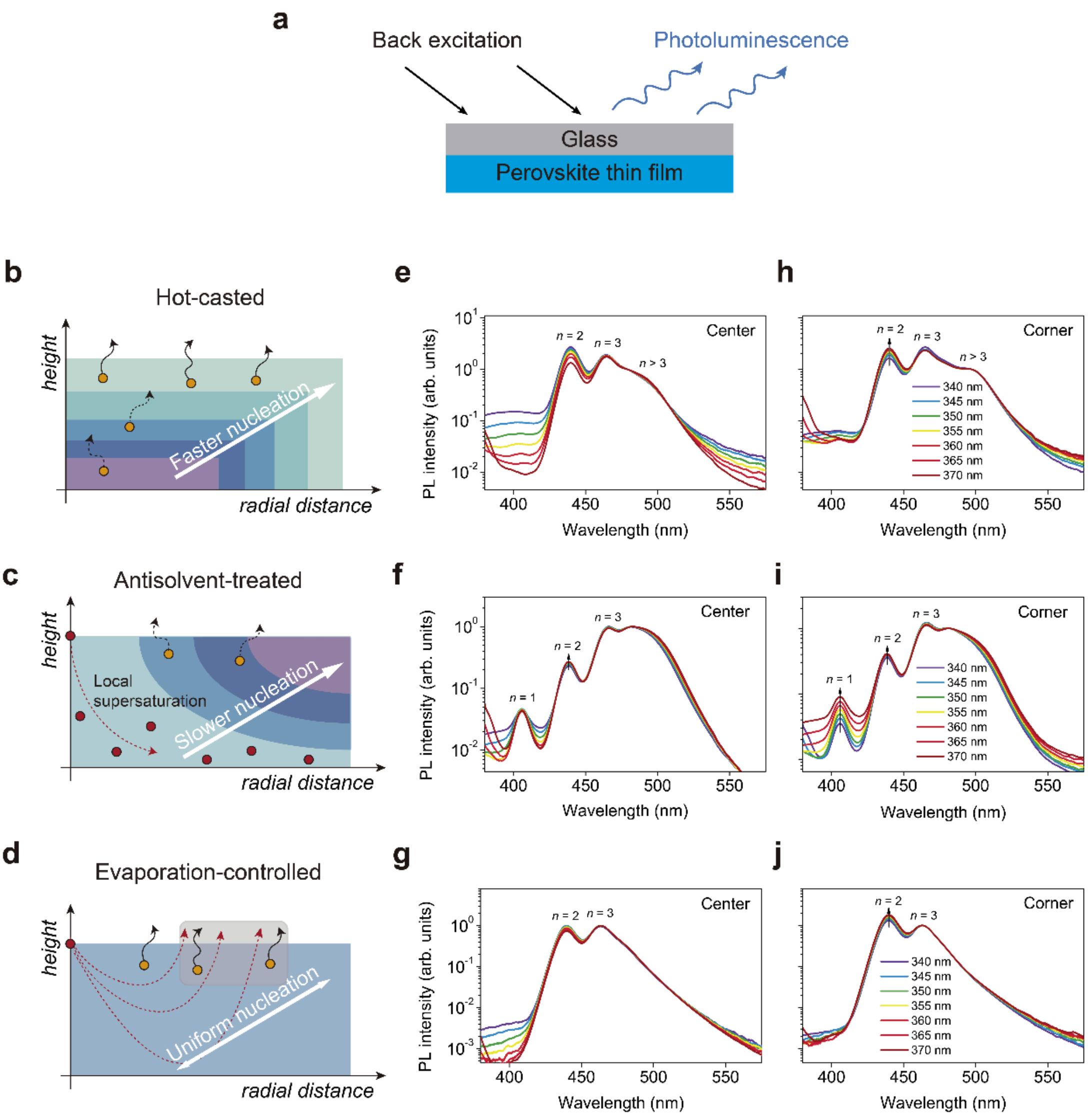


**Figure S1.** Phase distribution of <*n*> = 2 perovskites ($PEA_2CsPb_2Br_7$) synthesized using different techniques. a) Schematic diagram illustrating the photoluminescence measurement setup. Quasi-2D perovskites were illuminated from the back side of the films under various excitation wavelengths. b-d) Schematic diagrams illustrating the phase distribution of perovskites. e-g) Normalized photoluminescence spectra of center and h-j) corner area of the films measured with different excitation wavelengths.

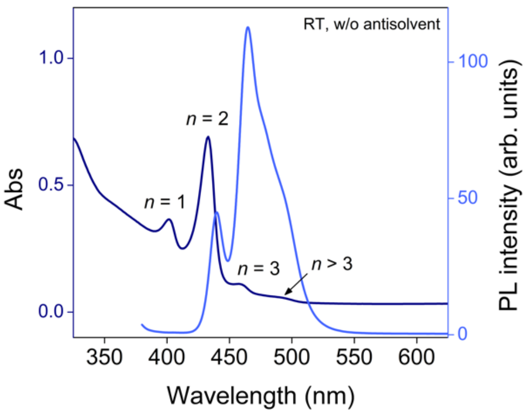


**Figure S2.** Photophysical properties of <*n*> = 2 perovskites ($PEA_2CsPb_2Br_7$) synthesized without the addition of antisolvent. The excitation wavelength was 365 nm.

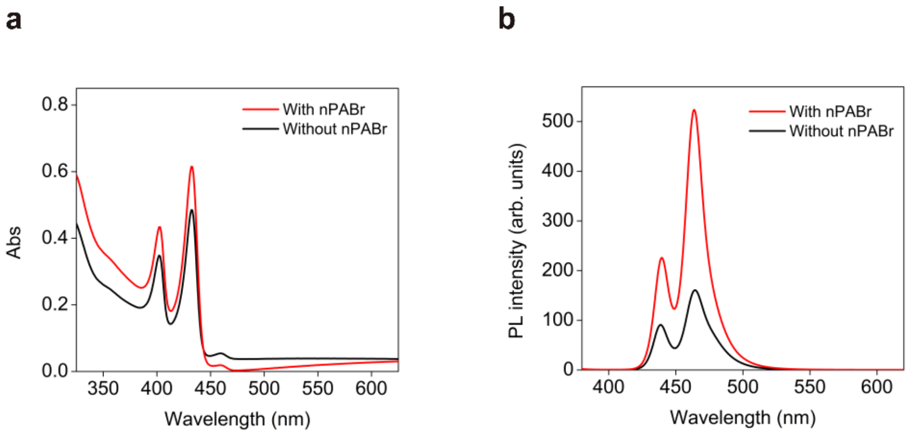


**Figure S3.** Photophysical properties of evaporation-controlled <*n*> = 2 perovskites ($PEA_2CsPb_2Br_7$) synthesized with and without the addition of nPABr. The excitation wavelength was 365 nm.

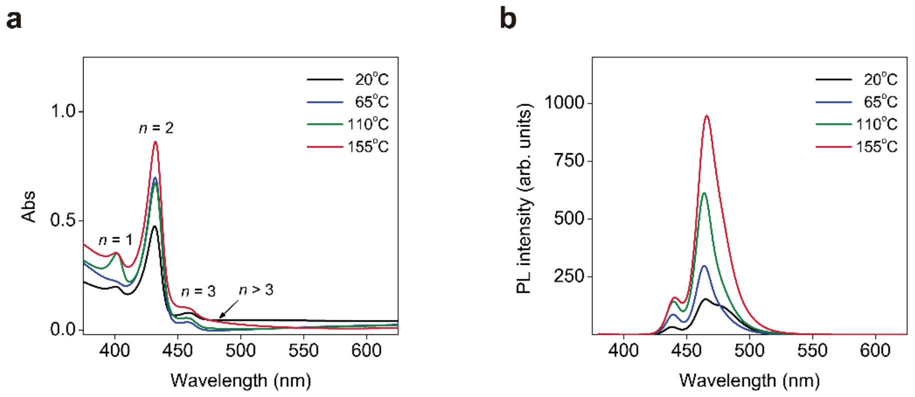


**Figure S4.** Temperature dependence. a) UV-Vis absorption and b) photoluminescence spectra of <*n*> = 2 perovskites ($PEA_2CsPb_2Br_7$) spin-coated at various temperatures. During the synthesis, 150 µL of toluene (boiling point: 110.6°C) was dropped onto the substrate. The excitation wavelength was 365 nm.

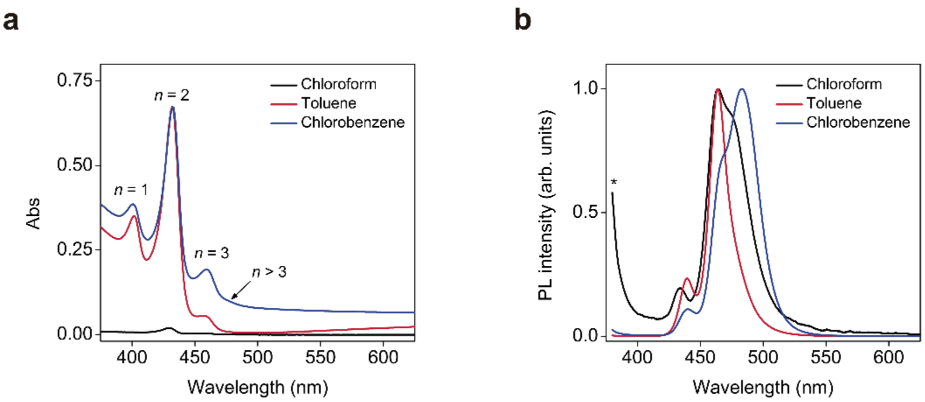


**Figure S5.** Antisolvent dependence. a) UV-Vis absorption and b) photoluminescence spectra of <*n*> = 2 perovskites ($PEA_2CsPb_2Br_7$) synthesized with various antisolvents. The excitation wavelength was 365 nm. The peak marked with an asterisk (*) corresponds to the excitation beam.

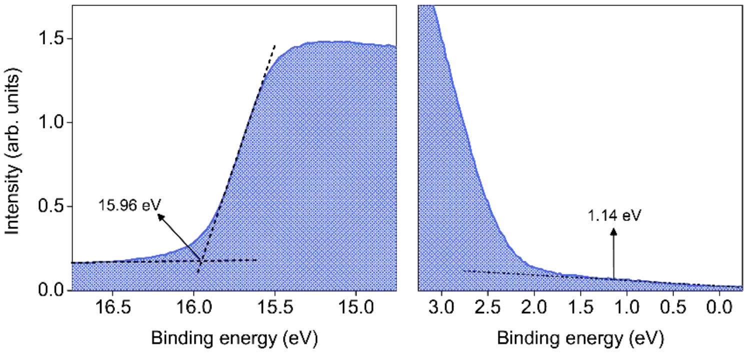


**Figure S6.** Ultraviolet photoelectron spectrum of an <*n*> = 2 perovskite ($PEA_2CsPb_2Br_7$) spin-coated on the ITO substrate.

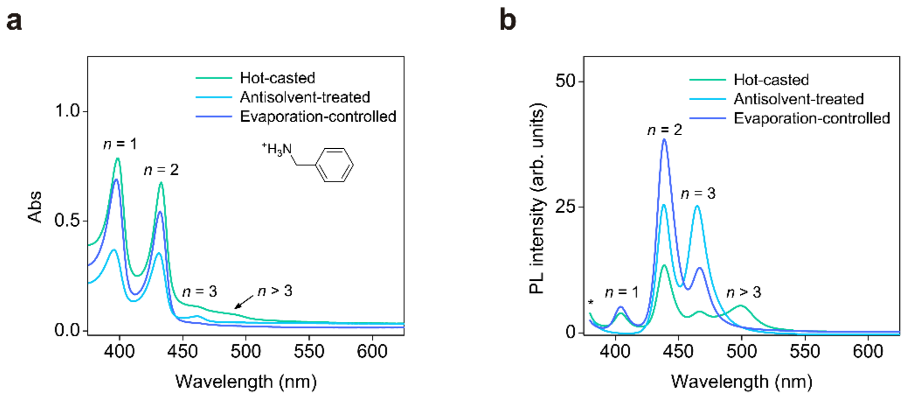


**Figure S7.** Photophysical characterizations of BA-based <*n*> = 2 perovskites synthesized using different techniques. a) UV-vis absorption and b) photoluminescence spectra of $BA_2CsPb_2Br_7$ perovskites. The excitation wavelength was 365 nm. The peak marked with an asterisk (*) corresponds to the excitation beam.

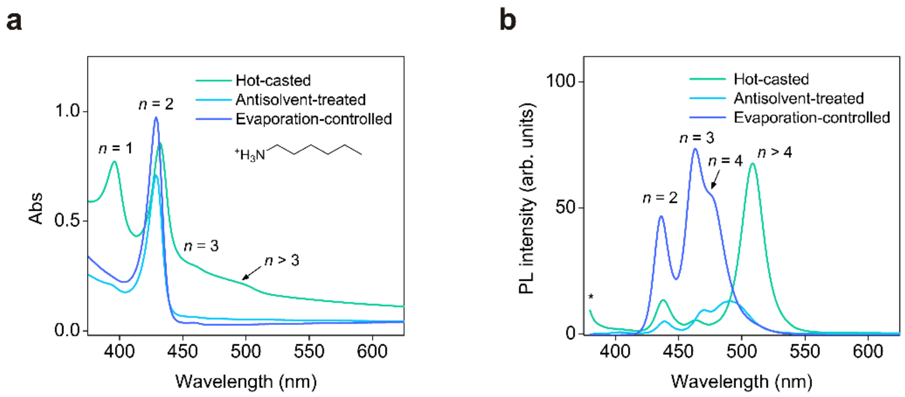


**Figure S8.** Photophysical characterizations of nHA-based <*n*> = 2 perovskites synthesized using different techniques. a) UV-vis absorption and b) photoluminescence spectra of $nHA_2CsPb_2Br_7$ perovskites. The excitation wavelength was 365 nm. The peak marked with an asterisk (*) corresponds to the excitation beam.

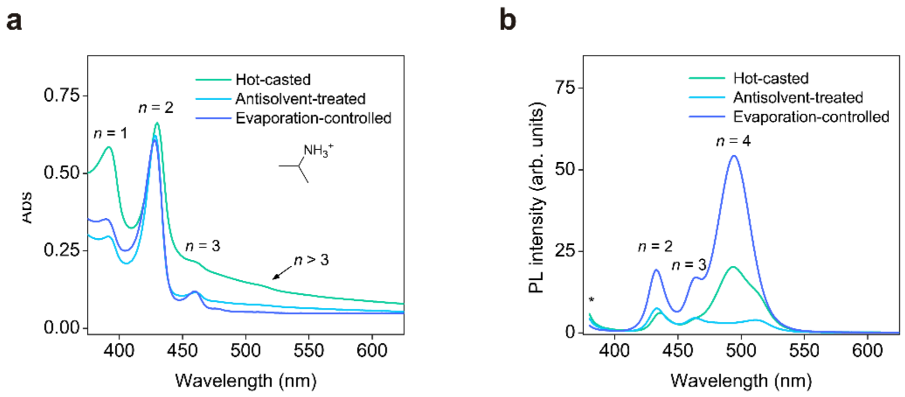


**Figure S9.** Photophysical characterizations of IPA-based <*n*> = 2 perovskites synthesized using different techniques. a) UV-vis absorption and b) photoluminescence spectra of $IPA_2CsPb_2Br_7$ perovskites. The excitation wavelength was 365 nm. The peak marked with an asterisk (*) corresponds to the excitation beam.

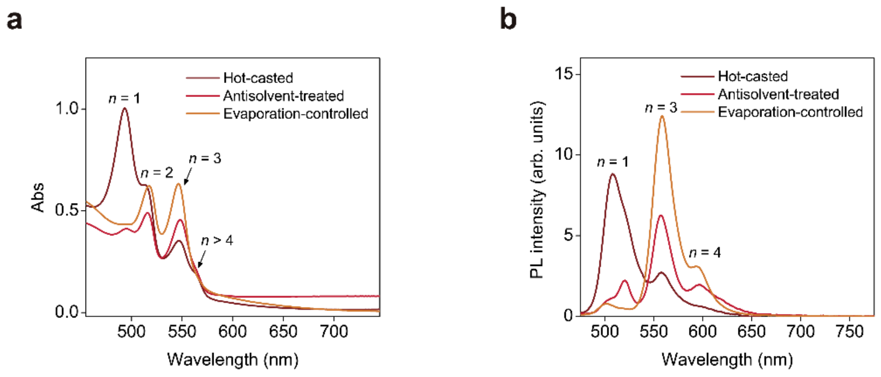


**Figure S10.** Photophysical characterizations of iodide-based <*n*> = 2 perovskites synthesized using different techniques. a) UV-vis absorption and b) photoluminescence spectra of $PEA_2CsPb_2I_7$ perovskites. The excitation wavelength was 365 nm.

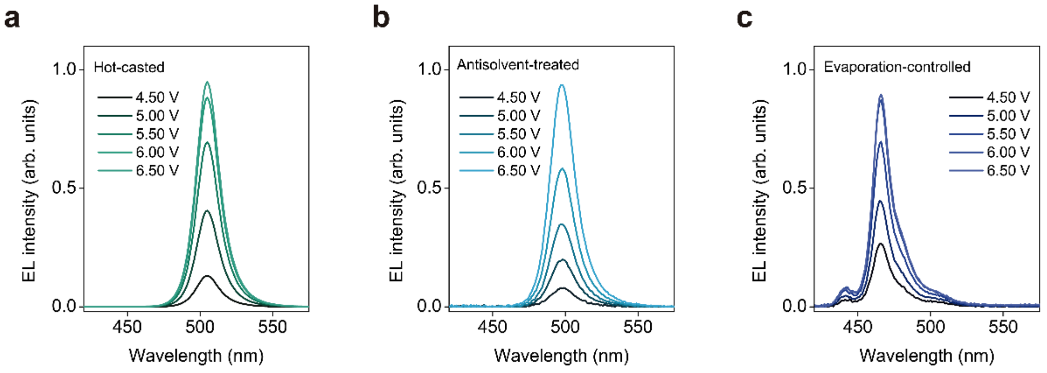


**Figure S11.** Spectral stability. Electroluminescence spectra of a) hot-casted, b) antisolvent-treated, and c) evaporation-controlled <*n*> = 2 PEA-based perovskites measured at various driving voltages.

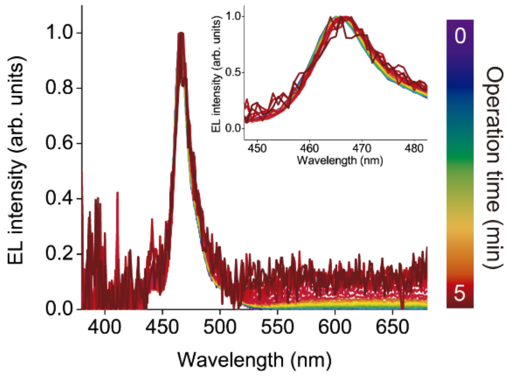


**Figure S12.** Normalized electroluminescence spectra of evaporation-controlled perovskites over time demonstrating the operational stability. The driving voltage was 6.5 V.

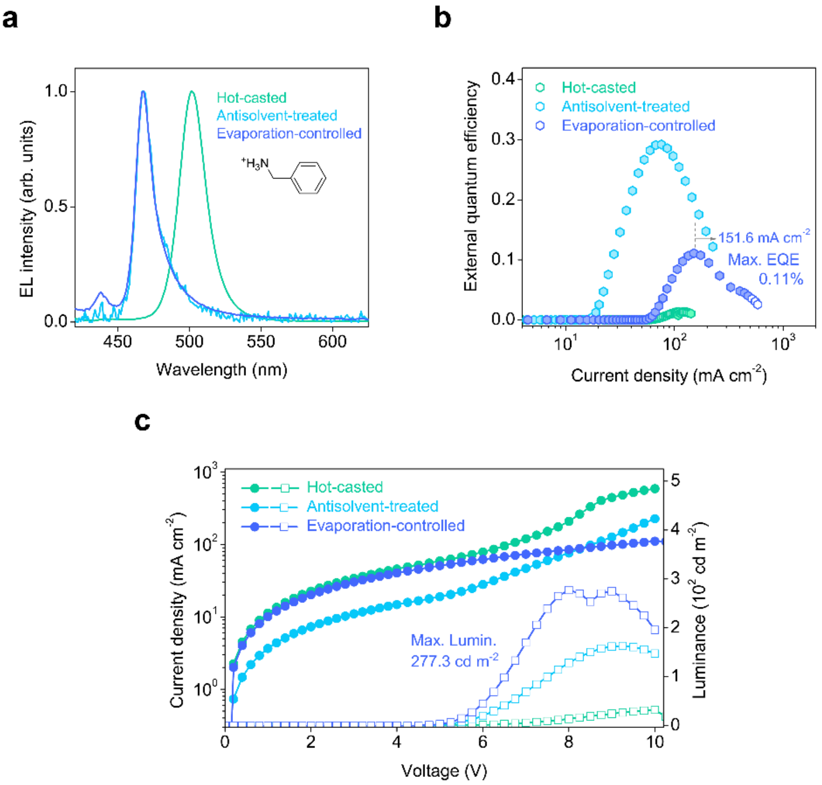


**Figure S13.** Device performance of BA-based <$n$> = 2 perovskites synthesized using different techniques. a) Electroluminescence spectrum of the device measured at a driving voltage of 7.0 V. b) Plots of external quantum efficiency as a function of current density. c) Plots of current density and luminance as a function of driving voltage.

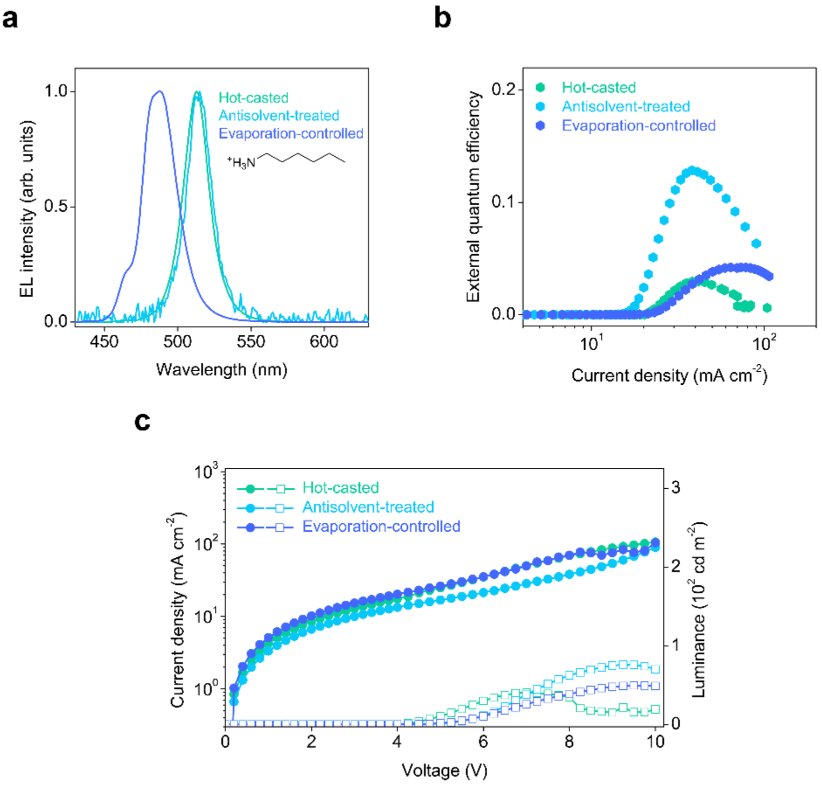


**Figure S14.** Device performance of nHA-based <*n*> = 2 perovskites synthesized using different techniques. a) Electroluminescence spectrum of the device measured at a driving voltage of 7.0 V. b) Plots of external quantum efficiency as a function of current density. c) Plots of current density and luminance as a function of driving voltage.

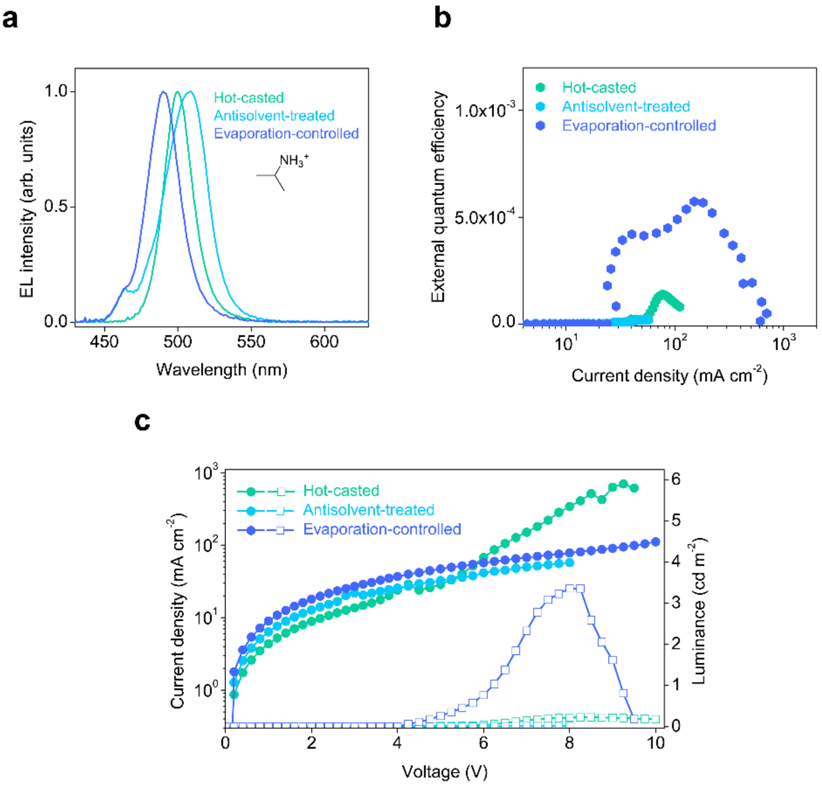


**Figure S15.** Device performance of iPA-based <*n*> = 2 perovskites synthesized using different techniques. a) Electroluminescence spectrum of the device measured at a driving voltage of 7.0 V. b) Plots of external quantum efficiency as a function of current density. c) Plots of current density and luminance as a function of driving voltage.

**Table S1.** Device performance of reported deep-blue-emissive LEDs based on quasi-2D perovskites.

| Institution | Year | Material | $\lambda_{EL}$ (nm) | FWHM (nm) | CIE (x,y) | Luminance$_{max}$ (cd m$^{-2}$) | J$_{roll-off}$ (mA cm$^{-2}$) | Ref |
|---|---|---|---|---|---|---|---|---|
| Soochow University | 2019 | $(P\text{-}PDA_{0.75}/PEA_{0.25})_2CsPb_2Br_7$ | 465 | 25 | (0.14, 0.05) | 211.0 | <1 | 1 |
| Nanyang Technological University | 2020 | $PBA_{3.33}CsPb_2Br_{7.33}$ | 465 | ~25 | (0.13, 0.08) | 144.9 | <0.01 | 2 |
| University of California, Berkeley | 2020 | $BA_2Cs_{n-1}Pb_nBr_{3n+1}$ | 450 | 27.5 | - | - | ~10 | 3 |
| The University of Hong Kong | 2020 | $(BA_{0.5}PEA_{0.5})_2MAPb_2Br_7$ | 456 | - | (0.16, 0.09) | - | - | 4 |
| Kyung Hee University | 2021 | $PEA_2Cs_{n-1}Pb_nBr_{3n+1}$ | 464 | 27 | (0.15, 0.08) | 4.5 | ~1 | 5 |
| Ewha Womans University ***(This work)*** | 2022 | $PEA_2CsPb_2Br_7$ | 466 | 14 | (0.15, 0.10) | 240.0 | 126.7 | - |
| Ewha Womans University ***(This work)*** | 2022 | $BA_2CsPb_2Br_7$ | 467 | 14 | (0.15, 0.13) | 277.3 | 151.6 | - |

References

[1] S. Yuan, Z. K. Wang, L. X. Xiao, C. F. Zhang, S. Y. Yang, B. B. Chen, H. T. Ge, Q. S. Tian, Y. Jin, L. S. Liao, *Adv. Mater.* **2019**, *31*, 1.

[2] N. Yantara, N. F. Jamaludin, B. Febriansyah, D. Giovanni, A. Bruno, C. Soci, T. C. Sum, S. Mhaisalkar, N. Mathews, *ACS Energy Lett.* **2020**, *5*, 1593.

[3] H. Chen, J. Lin, J. Kang, Q. Kong, D. Lu, J. Kang, M. Lai, L. N. Quan, Z. Lin, J. Jin, L. Wang, M. F. Toney, P. Yang, *Sci. Adv.*, **2020**, *6*, 1.

[4] T. L. Leung, H. W. Tam, F. Liu, J. Lin, A. M. C. Ng, W. K. Chan, W. Chen, Z. He, I. Lončarić, L. Grisanti, C. Ma, K. S. Wong, Y. S. Lau, F. Zhu, Ž. Skoko, J. Popović, A. B. Djurišić, *Adv. Opt. Mater.* **2020**, *8*, 1.

[5] S. Kang, S. Park, S. Park, H. Kwon, J. Lee, K.-H. Hong, Y.-J. Pu, J. Park, *Mater. Today Energy*, **2021**, *21*, 1.
